# ARF6: Biomarker, Therapeutic Target, and AI-Driven Precision Management for Type 2 Diabetes with Obesity

**DOI:** 10.3390/metabo16100769

**Published:** 2026-10-12

**Authors:** Yangyang Wang, Ye Chen, Huiyan Wang

**Affiliations:** Jilin Collaborative Innovation Center for Antibody Engineering, Jilin Medical University, Jilin 132013, China

**Keywords:** ARF6, biomarker, type 2 diabetes, obesity, artificial intelligence, precision management, clinical translation

## Abstract

Type 2 diabetes combined with obesity is a prevalent metabolic comorbidity with high clinical heterogeneity, leading to inconsistent treatment efficacy and difficult long-term standardized management. Emerging evidence indicates that the small GTPase ADP-ribosylation factor 6 (ARF6) signaling axis plays a vital regulatory role in glycolipid metabolism disorders, insulin resistance and adipose tissue inflammation. Aberrant ARF6 expression is closely correlated with the occurrence and progression of type 2 diabetes and obesity, making it a promising pre-clinical candidate biomarker and potential druggable therapeutic target, which still requires large-scale clinical validation. Traditional clinical evaluation and intervention rely on conventional biochemical indicators and empirical treatment, which cannot meet the demands of individualized precision management. Artificial intelligence technology has gradually matured in endocrinology clinical practice, supporting accurate disease stratification, auxiliary diagnosis and optimized therapeutic decision-making. This review summarizes the pre-clinical significance of ARF6 in diabetic obesity and systematically discusses conceptual application prospects as well as major unresolved limitations of artificial intelligence in ARF6-oriented precision diagnosis and targeted intervention, aiming to provide practical references for the clinical translational research of metabolic precision management.

## 1. Introduction

The global prevalence of type 2 diabetes and obesity continues to rise, forming a severe public health burden across all age groups [1,2]. Diabetic obesity presents high clinical heterogeneity. Patients exhibit distinct degrees of insulin resistance, adipose inflammation and glycemic fluctuation [3]. These differences lead to inconsistent treatment efficacy and increased complication risks. Conventional clinical management mainly depends on body mass index, fasting blood glucose and glycosylated hemoglobin [4]. These routine indicators cannot fully capture the underlying molecular heterogeneity of metabolic disorders and may be insufficient to support accurate individual risk stratification and tailored treatment. Small GTPase ARF6 is a vital regulator of membrane trafficking, cytoskeleton remodeling and metabolic signal transduction [5,6]. Recent high-level pre-clinical studies together with limited preliminary human observations suggest a regulatory role of ARF6 in glycolipid metabolic homeostasis [7,8]. Aberrant ARF6 activation or downregulation has been associated with pancreatic β-cell dysfunction, peripheral insulin resistance and visceral adipose tissue remodeling [7,8]. Several pre-clinical studies indicate that aberrant ARF6 expression may represent a molecular signature linked to progressive T2DM and obesity [9]. In limited sample cohorts, it may help to distinguish metabolic severity and predict disease progression, although large-scale clinical validation remains absent, showing great potential as a novel non-invasive candidate biomarker and therapeutic target.

**Figure 1:**
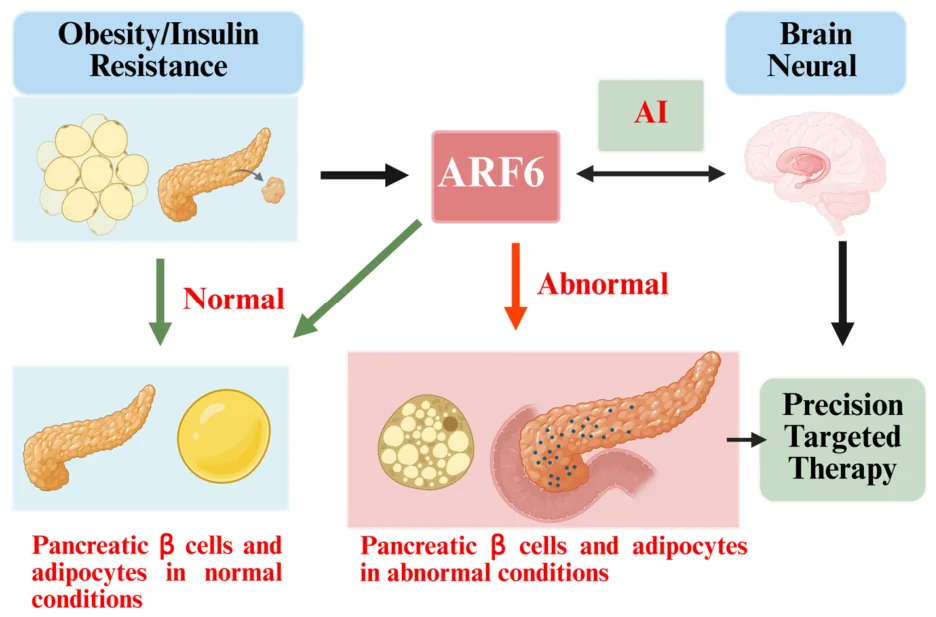
Regulatory role of ARF6 in type 2 diabetes with obesity and AI-driven precision targeted therapy pathway. This figure illustrates the role of ARF6 in obesity/insulin resistance: Normal ARF6 expression maintains the homeostasis of pancreatic β-cells and adipocytes, whereas abnormal expression leads to cellular dysfunction; by comparing the cellular phenotypes of pancreatic β-cells and adipocytes under physiological versus abnormal ARF6 conditions, this figure elucidates the biological basis for developing ARF6-targeted therapies combined with artificial intelligence. The neural elements in the figure represent hypothetical potential research directions; the central nervous-system-related mechanisms of ARF6 will not be discussed further in this review.

In recent years, artificial intelligence technology has achieved breakthrough applications in endocrinology and metabolic diseases. Machine learning and predictive modeling tools can mine high-dimensional clinical and molecular data. They realize intelligent auxiliary diagnosis, individualized risk assessment and precise treatment optimization for heterogeneous metabolic populations [10]. Compared with traditional empirical diagnosis, AI-driven strategies significantly improve the accuracy of disease stratification and avoid over-treatment or under-treatment [11]. Nevertheless, few review articles have systematically discussed the combined clinical potential of the ARF6 biomarker together with AI-enabled precision management for type 2 diabetes complicated with obesity. Most existing studies only discuss the basic mechanism of ARF6 or the broad application of AI in diabetes separately [9,12]. For this narrative review, we searched PubMed and Web of Science using keywords: ARF6, ADP-ribosylation factor 6, type 2 diabetes, obesity, insulin resistance, adipose inflammation, artificial intelligence, and precision management. Publications from Jan 2018 to May 2026 were screened. After initial database retrieval, title-and-abstract screening excluded records without thematic relevance. Potentially eligible papers proceeded to full-text assessment. Original research articles and review articles closely related to our theme were included; articles without direct relevance to ARF6, type 2 diabetes obesity or AI-enabled metabolic management were excluded. Reference lists of included papers were manually checked to capture additional relevant publications. This review integrates the latest evidence of ARF6 in regulating T2DM and obesity pathogenesis [9,13] (Figure 1). It focuses on the clinical transformation value of ARF6 as a novel candidate biomarker and therapeutic target for diabetic obesity. It further explores the application prospects and existing limitations of AI in ARF6-based precision diagnosis and targeted intervention. The purpose is to provide a new theoretical basis for improving the individualized clinical management system of diabetic obesity (Figure 1).

## 2. Biological Function of ARF6 in Glycolipid Metabolism and Metabolic Homeostasis

**Figure 2:**
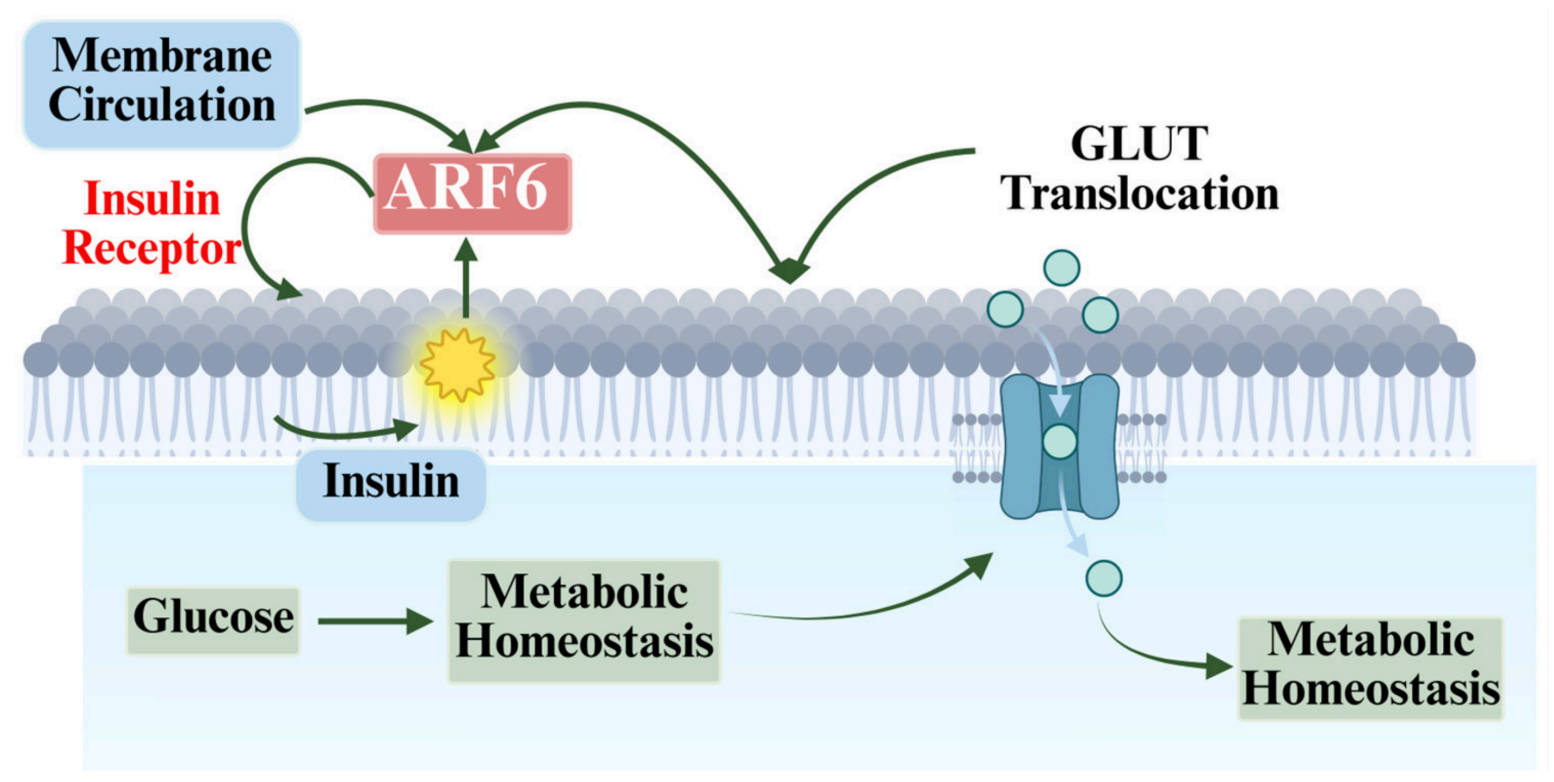
ARF6 regulates insulin signaling and glucose metabolic homeostasis through membrane trafficking. Insulin stimulation can activate ARF6, which promotes glucose uptake in peripheral tissues and maintains metabolic homeostasis by regulating the membrane cycling of insulin receptors and the membrane translocation of glucose transporters (GLUTs).

ARF6 is a conserved small GTP-binding protein widely expressed in metabolic key tissues, including pancreas, liver, skeletal muscle and adipose tissue [9,14] (Figure 2). It mainly regulates cell membrane transport, lipid metabolism and inflammatory signal activation. Its expression level and activation state are closely linked to the stability of systemic glycolipid metabolism [14,15,16]. Under physiological conditions, ARF6 maintains normal insulin signal transduction [9,17]. It promotes insulin receptor membrane recycling and glucose transporter translocation [9,13]. It contributes to efficient glucose uptake and utilization in peripheral tissues [7].

**Figure 3:**
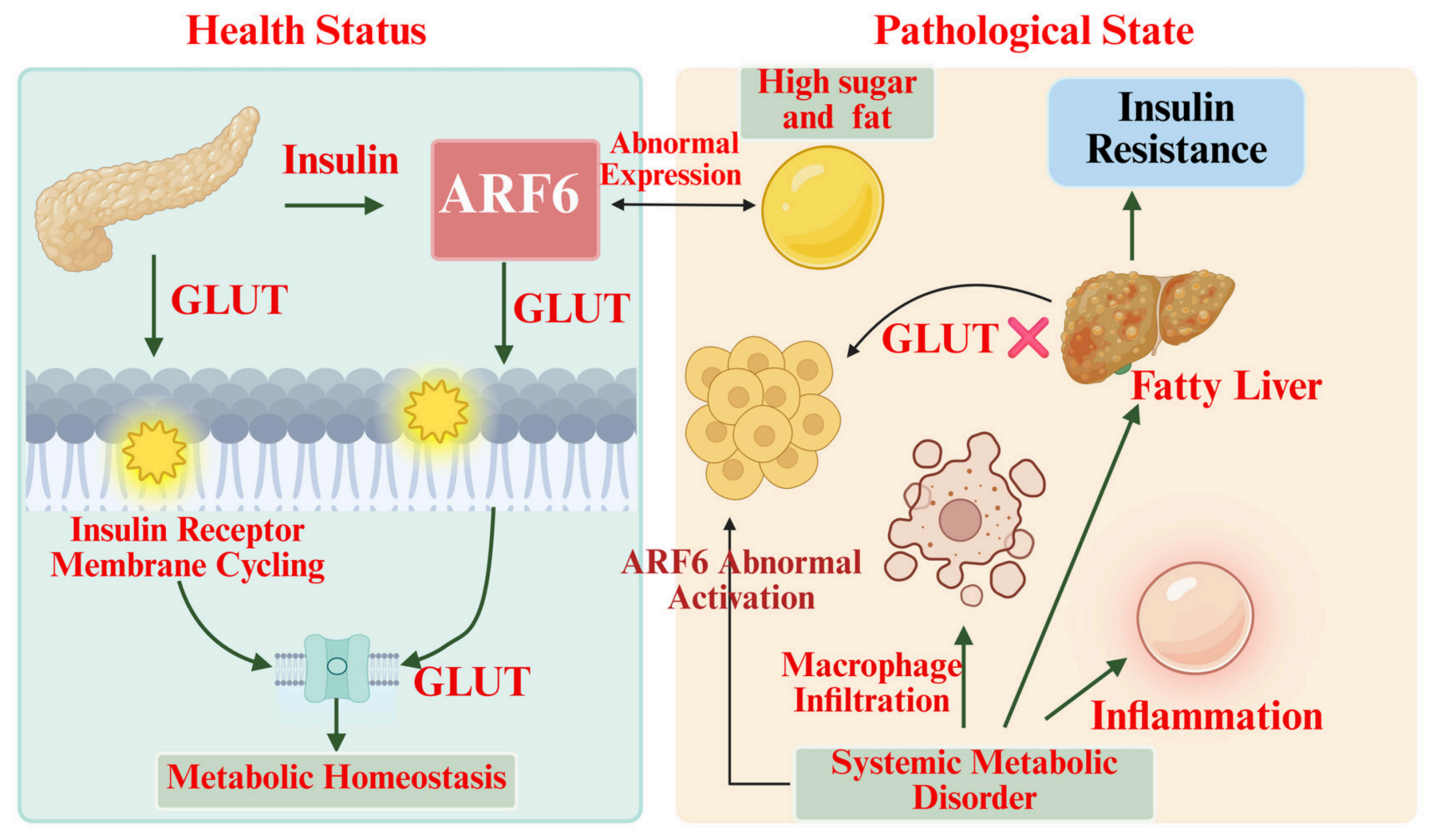
Schematic diagram of the mechanism by which ARF6 signaling dysregulation mediates systemic metabolic disorders under obesity and hyperglycemic conditions. Under physiological conditions, ARF6 maintains metabolic homeostasis; under obesity and hyperglycemic conditions, high sugar and high fat loads induce ARF6 signaling dysregulation, triggering insulin resistance and adipose inflammation, ultimately leading to systemic metabolic disorder.

Under obese and hyperglycemic conditions, ARF6 signaling is significantly dysregulated. High glucose and lipid overload induce abnormal ARF6 expression [7,18]. This condition impairs insulin signal conduction in skeletal muscle and liver, resulting in peripheral insulin resistance [19]. In adipose tissue, abnormal ARF6 activation promotes adipose macrophage infiltration and metabolic inflammation [20]. It accelerates visceral fat accumulation and adipose tissue fibrosis, further exacerbating systemic metabolic disorders [7]. Preliminary observations from limited clinical samples suggest that circulating ARF6 may correlate positively with BMI, fasting blood glucose and the insulin-resistance index and negatively with islet β-cell function [9,13]. It is worth noting that evidence from tissue-specific genes indicates that downregulation of ARF6 in endothelial cells alone can induce systemic insulin resistance, suggesting that ARF6 possesses differentiated metabolic functions that are highly dependent on the tissue microenvironment [7]. These findings strongly suggest that ARF6 is closely involved in the whole progression of diabetic obesity (Figure 3).

In diabetic complication models, ARF6 dysregulation also mediates renal podocyte cholesterol deposition and vascular endothelial dysfunction. It promotes the occurrence of diabetic microvascular complications [7,18,21]. This multi-tissue regulatory feature makes ARF6 different from traditional single metabolic indicators [22]. It can reflect the overall metabolic disorder degree and complication risk of patients, laying a foundation for its clinical biomarker application.

## 3. ARF6 as a Novel Biomarker and Therapeutic Target for Diabetic Obesity

### 3.1. Clinical Biomarker Value of ARF6

Traditional diagnostic indicators for diabetic obesity have obvious limitations. BMI cannot distinguish fat distribution and metabolic inflammation degree [23,24]. Glycosylated hemoglobin only reflects short-term average glycemic level [25]. They lack molecular specificity for early disease prediction and risk stratification. With the progress of translational metabolomics and proteomics, tissue-specific molecular biomarkers have become the new direction of metabolic precision diagnosis [26,27].

ARF6 shows potential advantages as a candidate biomarker for diabetic obesity (Table 1). Firstly, altered ARF6 expression arises early during metabolic disturbance and may predict progression from simple obesity toward hyperglycemia [7]. Secondly, in small cohorts, circulating ARF6 levels appear to correlate with insulin-resistance severity and adipose inflammation, which might help stratify metabolic heterogeneity among patients with similar conventional biochemical indicators [9,20]. Thirdly, ARF6 detection is minimally invasive. It may have broad prospects for clinical adoption, although its analytical performance still requires systematic clinical validation.

### 3.2. Targeted Therapeutic Potential of ARF6

Based on pre-clinical mechanistic evidence, ARF6 represents a potential druggable therapeutic target for diabetic obesity [9,30]. Specific inhibition of pathological ARF6 over-activation within metabolic tissues can reduce adipose-tissue inflammation [9]. Of note, endothelial-specific ARF6 depletion exerts opposing metabolic effects, as described in reference [7]. Although tissue-specific delivery strategies targeting ARF6 may reduce systemic side effects [7,9], these observations remain limited to pre-clinical models, and substantial barriers persist before clinical translation. In particular, non-selective ARF6 inhibition carries tissue-specific risks. Evidence from transgenic animals indicates that the absence of ARF6 in endothelial cells induces systemic insulin resistance, and non-selective ARF6 inhibition may also impair physiological insulin secretion by pancreatic β-cells [7,9,31]. These risks underscore the necessity of adopting a tissue-selective delivery strategy that inhibits ARF6 only in metabolically diseased tissues while preserving its homeostatic physiological functions in endothelial cells and β-cells.

**Figure 4:**
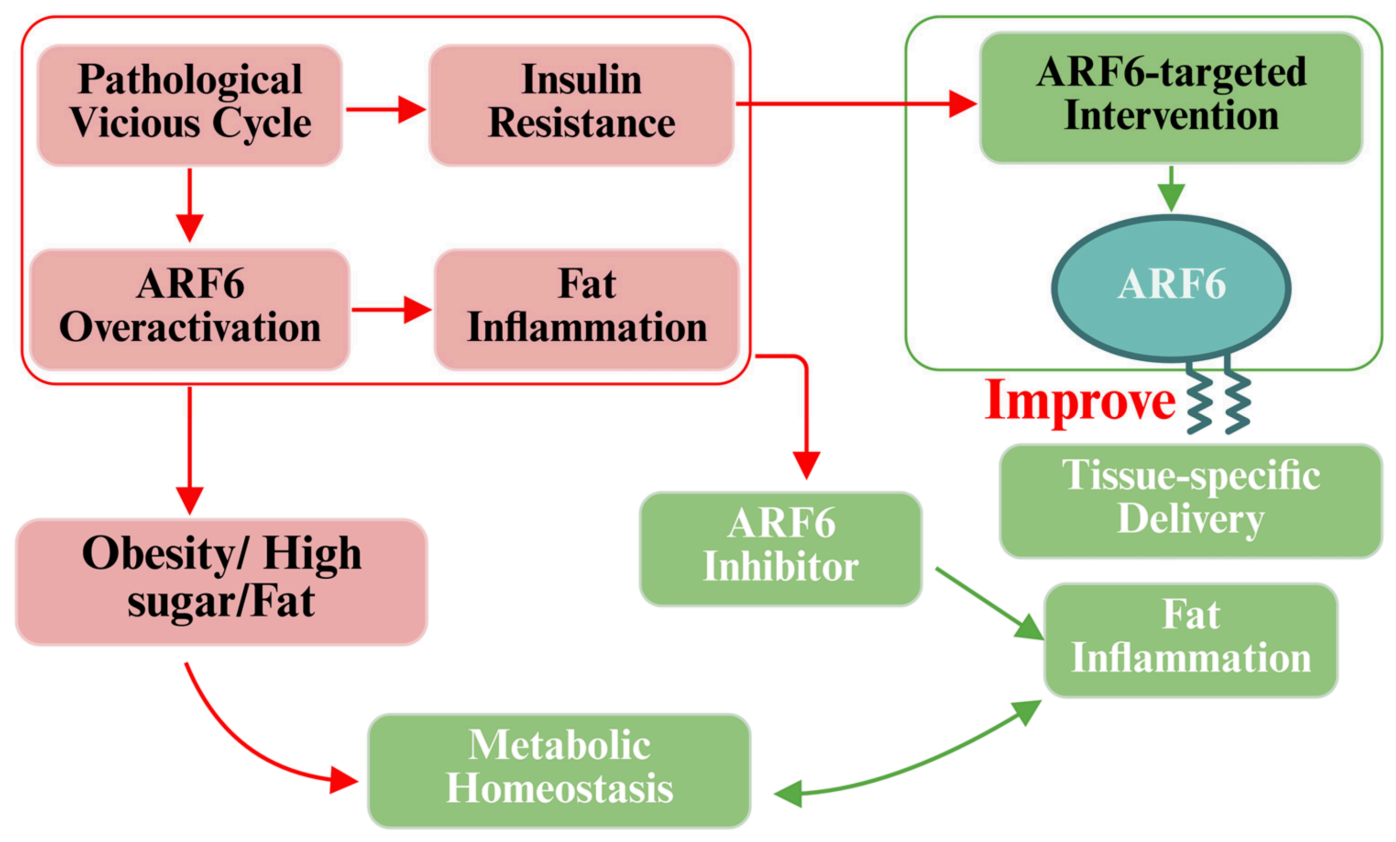
Proposed mechanism for ARF6-targeted intervention for diabetic obesity. In diabetic obesity, pathological ARF6 over-activation drives a vicious pathological cycle of insulin resistance and adipose inflammation. Targeted ARF6 inhibition combined with tissue-specific delivery can interrupt this cycle in pre-clinical models; substantial barriers remain before clinical translation can be realized.

Compared with existing metabolic targets, ARF6 has unique advantages. It acts on the upstream link of insulin signal and inflammatory response [9,13]. It can simultaneously improve insulin resistance and adipose inflammation and block the vicious cycle of metabolic disorder [7,9,20]. Therefore, ARF6-targeted therapy may offer prospective value for the individualized treatment of refractory diabetic obesity (Figure 4).

## 4. AI-Driven Precision Management Based on ARF6 Biomarker

This section describes a proposed conceptual framework for ARF6-oriented AI-driven precision management. To date, no patient-validated AI model combining ARF6 markers for diabetic obesity has been formally reported.

### 4.1. AI-Assisted Disease Risk Stratification and Early Diagnosis

The clinical heterogeneity of diabetic obesity makes traditional unified diagnosis standards unable to meet precision management needs. AI machine learning models have data mining capabilities [32,33]. They can integrate ARF6 molecular indicators with routine clinical data, imaging features and demographic information [14,34,35,36]. They may be used to construct disease risk prediction models [37].

**Figure 5:**
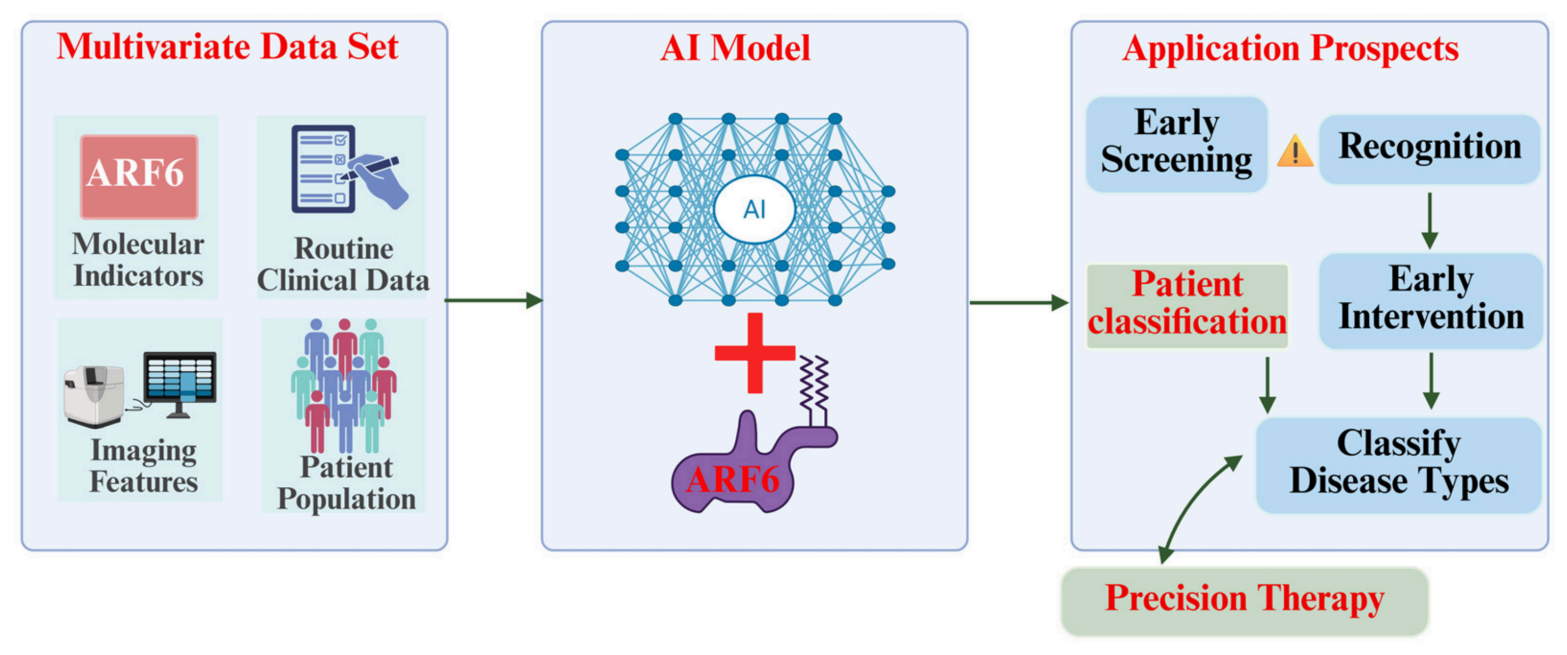
Conceptual diagram for AI-assisted early-diagnosis and risk-stratification workflows incorporating ARF6. Hypothetical AI models integrating ARF6 molecular markers alongside multi-dimensional clinical data could construct predictive pipelines that might support early-risk screening and patient molecular subtyping, if prospectively validated. The figure is schematic and does not display measured model outputs.

**Figure 6:**
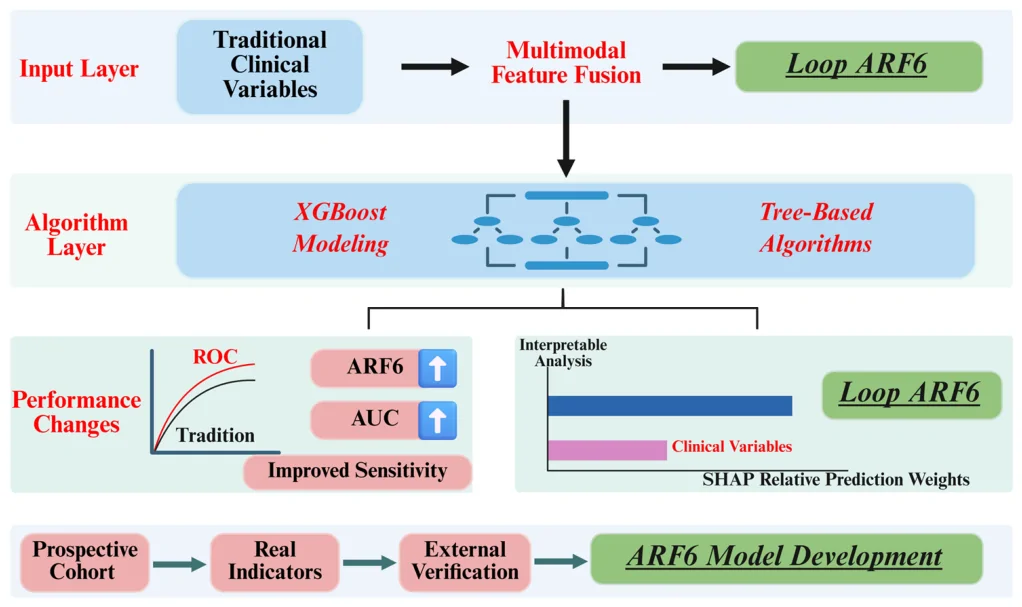
ARF6: Analytical paradigms combining machine learning and the current status of clinical validation. In this schematic, “Loop ARF6” represents an analytical workflow: biomarker features derived from ARF6 are input into a multimodal machine learning pipeline to perform iterative model building and evaluation. Published research on metabolic diseases indicates that, within a machine-learning-based analytical paradigm, tree-based ensemble algorithms such as XGBoost are well-suited for integrating circulating molecular biomarkers with traditional clinical variables. The figure is a conceptual diagram only and does not display any measured performance metrics, axis scales, or empirical SHAP weight data for ARF6. The lengths of the bars in the SHAP plot are used solely to illustrate the workflow and do not represent experimentally derived predictive weights when comparing ARF6 with clinical variables. SHAP-based feature importance analysis can further quantify the predictive weights of circulating molecular biomarkers in multimodal models, providing a reference framework for the future development and clinical validation of ARF6-related models [40].

Recent studies suggest that AI models based on multi-dimensional molecular biomarkers may improve the accuracy of early diagnosis of metabolic diseases [37,38,39]. By combining ARF6 expression levels with clinical indicators using AI algorithms, it may be possible to screen for individuals at high risk of progressing from simple obesity to type 2 diabetes; this approach may enable early warning and intervention for metabolic deterioration, and the proposed AI stratification model could classify patients with obesity and type 2 diabetes into different molecular subtypes based on ARF6 expression patterns [37]. In addition, AI-driven stratification models could subtype diabetic–obese patients according to ARF6-related molecular features, which might provide a basis for individualized treatment planning (Figure 5 and Figure 6).

### 4.2. AI-Optimized Individualized Targeted Intervention

Traditional treatment of diabetic obesity adopts unified diet, exercise and drug intervention schemes. It ignores individual molecular differences, resulting in poor treatment response in some patients [32,41]. In the future, based on ARF6 biomarker characteristics, artificial intelligence may have the capacity to intelligently optimize treatment strategies. For patients with high ARF6 expression and severe adipose inflammation, AI models could potentially recommend targeted anti-inflammatory and metabolic regulation schemes [9,42,43]. For patients dominated by insulin resistance caused by ARF6-mediated signal dysfunction, such models might prioritize insulin-sensitization intervention [7,9].

**Figure 7:**
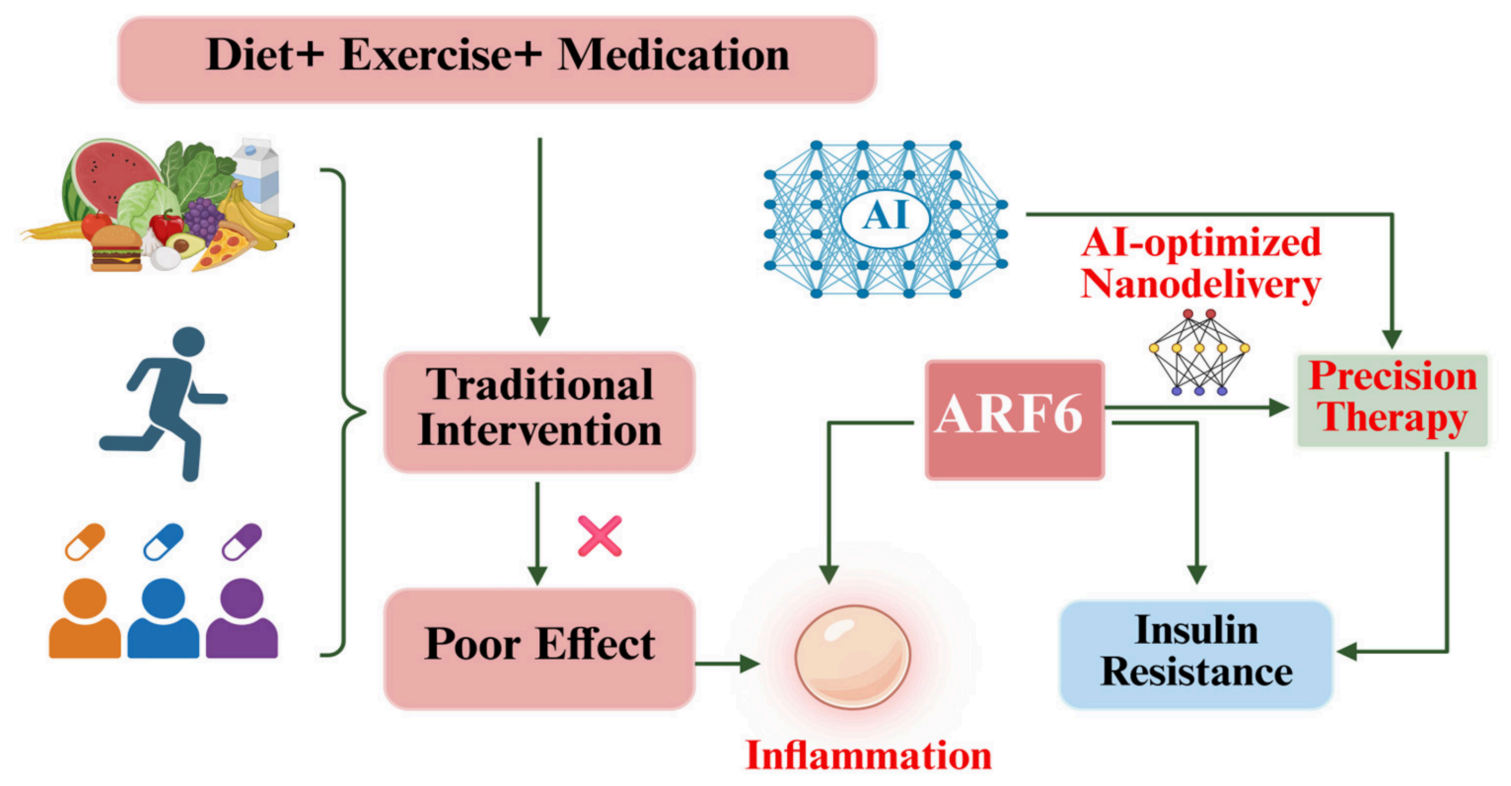
Personalized targeted intervention model for diabetic obesity based on AI and ARF6. Traditional intervention models largely ignore individual differences and often achieve poor effects. In this conceptual framework, AI could use ARF6-related characteristics to formulate differentiated treatment plans and optimize targeted delivery, potentially enabling more precise and personalized management of diabetic obesity.

In addition, it is anticipated that AI simulation technology can optimize ARF6-targeted nanodelivery parameters. It improves the tissue targeting efficiency and biosafety of targeted drugs [44,45], which may offer technical support for the future clinical translation of ARF6-targeted therapy [14]. This hypothetical intelligent-intervention paradigm could break the limitations of traditional empirical treatment and enable precise matching between molecular subtyping and clinical treatment (Figure 7).

### 4.3. AI-Based Long-Term Disease Monitoring and Prognosis Evaluation

Long-term dynamic monitoring is the key to reducing complications of diabetic obesity. Traditional regular re-examination cannot capture subtle molecular changes in the early stage of disease progression [46,47]. At present, clinically available technology for continuous or real-time measurement of circulating ARF6 is not yet established, which differs from well-developed continuous glucose-monitoring devices [47]. Therefore, real-time tracking of circulating ARF6 for complication risk prediction remains a hypothetical future technical vision. If such detection techniques could be developed in the future, AI-based intelligent monitoring systems might interpret serial, periodically measured circulating ARF6 data together with daily blood-glucose, body-weight and other metabolic data to evaluate trends in disease progression.

**Figure 8:**
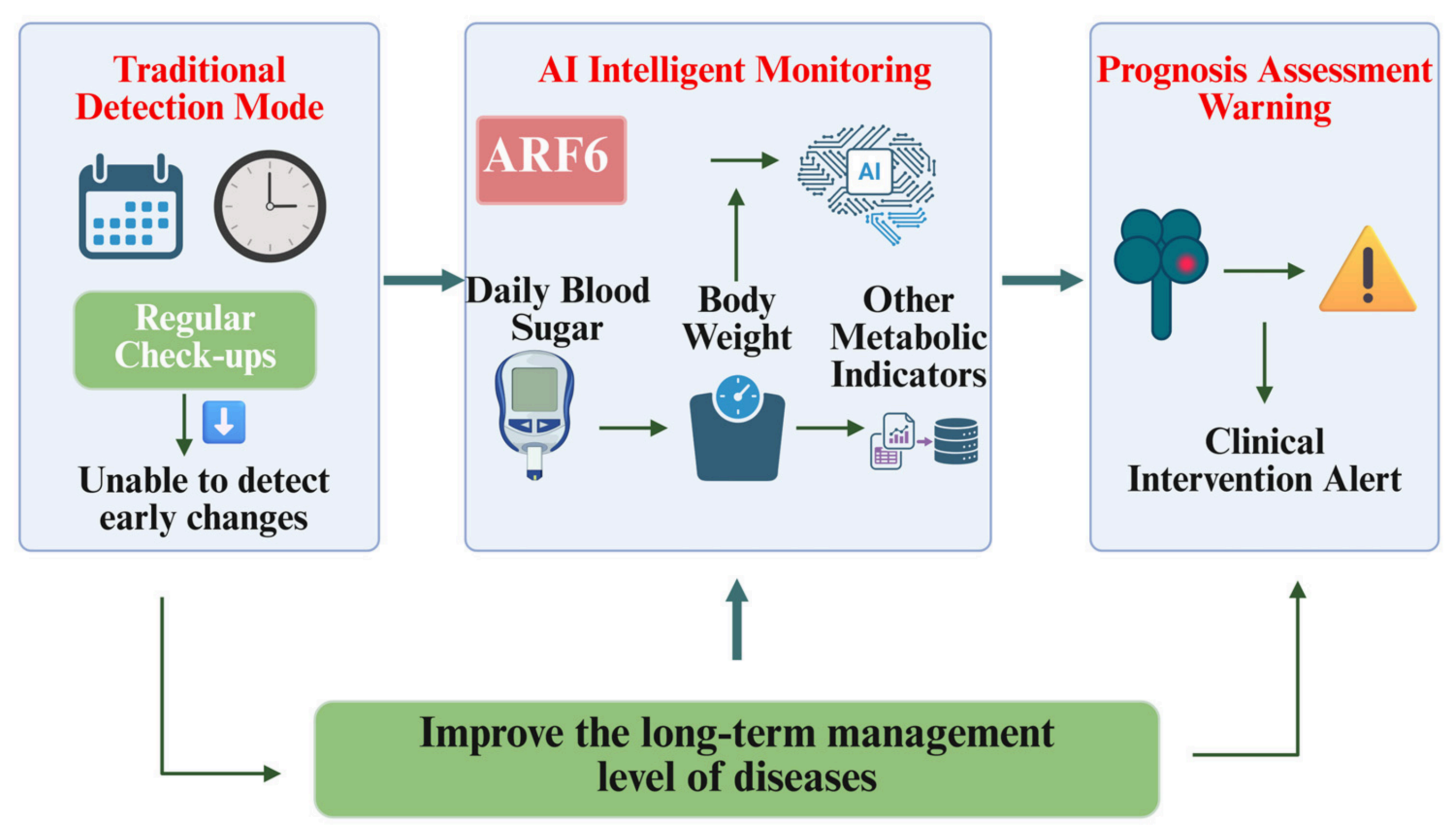
Hypothetical long-term monitoring and prognostic-assessment model for diabetic obesity based on ARF6 and artificial intelligence. Traditional periodic check-ups cannot readily capture early pathological shifts. This schematic illustrates a potential future workflow: if continuous ARF6 measurement technology were developed, AI systems integrating serial ARF6 read-outs together with daily metabolic data might enable complication-risk alerting and could improve long-term patient management.

Under this proposed conceptual framework, if serial ARF6 measurements became clinically available, AI prognostic models could assess the risk of diabetic nephropathy, vascular lesions and other complications based on serial changes in ARF6 [15,48]. Such a potential monitoring-and-warning system would compensate for the limitations of conventional static biomarkers and optimize long-term management for patients with diabetic obesity [49,50] (Figure 8).

## 5. Discussion

Diabetic obesity is a complex metabolic disease driven by multiple genes and environmental factors. Its clinical heterogeneity is the core difficulty restricting precise clinical management [32,51]. Traditional diagnostic and therapeutic strategies rely on macroscopic phenotypic indicators [32,52]. They cannot reflect the underlying molecular pathological characteristics of patients, resulting in low accuracy of risk stratification and poor individualization of treatment [27]. This review indicates that ARF6 is a core molecular regulator of glycolipid metabolism disorder in diabetic obesity [9,14]. Its abnormal expression is closely related to insulin resistance, adipose inflammation and disease progression, which can serve as a candidate biomarker and therapeutic target for diabetic obesity [7,16].

Compared with traditional metabolic biomarkers such as FGF21 and GDF15, preclinical evidence suggests that ARF6 exerts a relatively direct regulatory role in insulin signaling and adipose tissue remodeling. It is expected to reflect key molecular characteristics of metabolic disorders at the molecular level and has the potential for early disease assessment; however, there is currently a lack of accurate data from human studies [9,31,53,54,55]. The combination of ARF6 molecular markers and AI technology further expands its clinical application value [56,57]. AI models may help extract the relationship between ARF6 dynamic changes and clinical phenotypes. It may break through the limitations of single static detection and realizes intelligent stratification, accurate diagnosis and individualized intervention of diabetic obesity [7,58,59]. This cross integration of molecular biomarkers and artificial intelligence provides a new research direction for the precision management of metabolic diseases.

Despite the good application prospects, the clinical transformation of ARF6 and AI combined strategy still has some limitations. In terms of biomarker implementation, circulating ARF6 suffers from unresolved technical obstacles, including non-standardized detection assays, undefined normal reference intervals, and potential confounding effects from renal clearance, all of which hinder its direct translation into routine clinical practice. First, most current studies on ARF6 in diabetic obesity are preclinical basic experiments, and large-sample, multi-center clinical cohort studies are still insufficient. The cutoff value of circulating ARF6 for clinical diagnosis and risk stratification lacks a unified standard [13,57,60]. Second, most AI prediction models are based on single-center retrospective data. The generalization ability and clinical practicability of the models need to be further verified by prospective studies [27,61]. Third, the relatively high technical cost of AI-based intelligent monitoring and targeted intervention limits their popularization and application in primary medical institutions [62,63].

**Figure 9:**
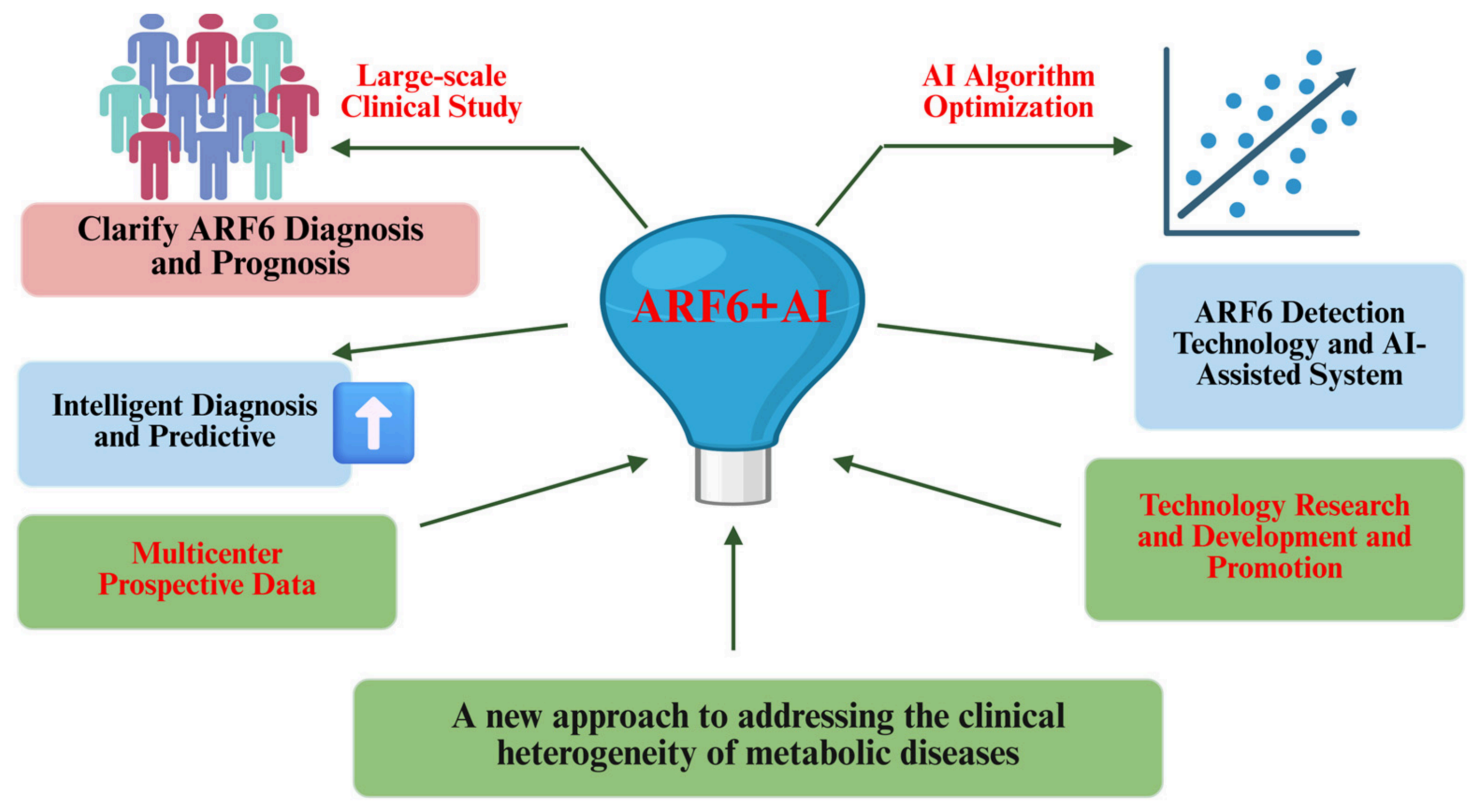
Future research directions of ARF6 combined with AI in precision management of diabetic obesity. Future research focus: clarify the diagnostic and prognostic criteria of ARF6, optimize AI algorithm models, and develop low-cost detection systems. ARF6 combined with AI is expected to become a new approach to addressing the clinical heterogeneity of metabolic diseases.

Future research should focus on three directions. First, carry out large-sample clinical studies to clarify the diagnostic threshold and prognostic evaluation standard of ARF6 in diabetic obesity. Second, optimize AI algorithm models based on multi-center prospective data to improve the accuracy and generalization of intelligent diagnosis and prediction. Third, develop low-cost ARF6 detection technology and simplified AI auxiliary diagnosis system. It promotes the popularization of precision management mode in grass-roots clinical practice. ARF6-centred biomarker strategies combined with artificial-intelligence workflows hold theoretical potential for improving clinical management of diabetic obesity (Figure 9). Further multi-center prospective research will be required before these approaches can be translated into real-world clinical tools.

## 6. Conclusions

ARF6 is a key regulatory molecule of glycolipid metabolism and adipose tissue homeostasis. Its abnormal expression represents an important molecular feature of type 2 diabetes combined with obesity. It shows promise as a pre-clinical candidate biomarker for early-warning and risk-stratification, as well as a potential pre-clinical therapeutic target, and unified clinical cutoff values and prospective cohort validations are still urgently needed.

## Figures and Tables

**Table 1:** Comparison of ARF6 and traditional diagnostic indicators in diabetic obesity. This table compares the advantages of ARF6 over traditional diagnostic indicators in early prediction and clinical application, while also covering metabolic inflammation, insulin-resistance assessment and patient-stratification performance.

Indicator Type	Traditional Indicators	ARF6	Limitations of ARF6
**Fat Distribution and Metabolic Inflammation**	BMI fails to distinguish fat distribution and the degree of metabolic inflammation [22,23,24]	Circulating ARF6 levels strongly correlate with fat inflammation severity and accurately reflect adipose tissue inflammatory status [9,20]	Evidence of the pathogenic effects of ARF6 is limited to mouse models; there is a lack of causal validation in large animals and clinical settings [7,9]
**Timeliness of Blood Glucose Level Reflection**	Glycated hemoglobin only reflects short-term average blood glucose levels [25]	Enables early warning of blood glucose changes during the transition from simple obesity to hyperglycemia [7]	Not suitable for systemic drug suppression, as it can lead to off-target effects such as endothelial damage and impaired insulin secretion [9,13]
**Molecular Specificity for Early Disease Prediction**	Lacks molecular specificity for early disease prediction and risk stratification of diabetes/obesity [26,27]	Exhibits abnormal expression in early metabolic disorders, providing predictive molecular specificity even before traditional indicators change [7]	ARF6 is not a molecule unique to glucose metabolism; it is also involved in multiple pathways, including vascular inflammation, the blood-brain barrier, and tumors, and is not specific to metabolic diseases [7]
**Assessment of Insulin Resistance Severity**	Cannot accurately assess the severity of a patient’s insulin resistance [9,20]	Circulating ARF6 levels strongly correlate with insulin resistance severity and accurately reflect its progression [9,20]	Local ARF6 deficiency in the endothelium can drive insulin resistance on its own and cannot be used as a standalone quantitative assessment metric [7]
**Patient metabolic heterogeneity**	Fails to stratify metabolic heterogeneity in patients with similar conventional biochemical profiles [9,20]	Enables precise stratification of metabolic heterogeneity in diabetic obese patients with similar conventional biochemical profiles [9,20]	ARF6 is merely a regulatory node for membrane transport, not a master switch for disease [9,13]
**Convenience and Safety of Testing Method**	Complex omics assays are difficult and operationally complex [26]	Simple, minimally invasive detection method; no complex omics testing required, ensuring high clinical feasibility [13,28]	There are detection biases between different test kits [14]
**The clinical translational value of the test results**	Poor reproducibility and high cost hinder large-scale clinical implementation [13,28,29]	Promising clinical-translation potential, though analytical performance and cost-effectiveness remain to be systematically verified in clinical cohorts [13,28,29]	ARF6-targeted drug inhibition strategies in vivo exhibit off-target effects and lack specificity [7,14]
**Industry Adaptability of Precision Metabolic Diagnostics**	No longer meets the clinical demand for precise diagnosis of modern metabolic diseases [26,27]	Organ-specific molecular biomarkers represent the core direction for advancing precise metabolic diagnostics [26,27]	There are currently no established tissue-selective targeting strategies for ARF6, and significant barriers to clinical translation remain [9,13]

## Data Availability

No new data were created or analyzed in this study.
